# Voices to reckon with: perceptions of voice identity in clinical and non-clinical voice hearers

**DOI:** 10.3389/fnhum.2013.00114

**Published:** 2013-04-03

**Authors:** Johanna C. Badcock, Saruchi Chhabra

**Affiliations:** ^1^School of Psychology, The University of Western AustraliaPerth, WA, Australia; ^2^Centre for Clinical Research in Neuropsychiatry, School of Psychiatry and Clinical Neurosciences, University of Western AustraliaPerth, WA, Australia

**Keywords:** hallucination, schizophrenia, voice perception, voice identity, voice recognition

## Abstract

The current review focuses on the perception of voice identity in clinical and non-clinical voice hearers. Identity perception in auditory verbal hallucinations (AVH) is grounded in the mechanisms of human (i.e., real, external) voice perception, and shapes the emotional (distress) and behavioral (help-seeking) response to the experience. Yet, the phenomenological assessment of voice identity is often limited, for example to the gender of the voice, and has failed to take advantage of recent models and evidence on human voice perception. In this paper we aim to synthesize the literature on identity in real and hallucinated voices and begin by providing a comprehensive overview of the features used to judge voice identity in healthy individuals and in people with schizophrenia. The findings suggest some subtle, but possibly systematic biases across different levels of voice identity in clinical hallucinators that are associated with higher levels of distress. Next we provide a critical evaluation of voice processing abilities in clinical and non-clinical voice hearers, including recent data collected in our laboratory. Our studies used diverse methods, assessing recognition and binding of words and voices in memory as well as multidimensional scaling of voice dissimilarity judgments. The findings overall point to significant difficulties recognizing familiar speakers and discriminating between unfamiliar speakers in people with schizophrenia, both with and without AVH. In contrast, these voice processing abilities appear to be generally intact in non-clinical hallucinators. The review highlights some important avenues for future research and treatment of AVH associated with a need for care, and suggests some novel insights into other symptoms of psychosis.

## Introduction

Auditory hallucinations usually involve hearing voices that no-one else can hear (Bentall, 2003). People with schizophrenia hear voices, and people without schizophrenia (or any other form of mental illness) hear voices, though the prevalence rates differ (Romme and Escher, 1989; Beavan et al., 2011; Kelleher et al., 2012). In fact, there is growing recognition of a number of other differences in auditory verbal hallucinations (AVH) in clinical and non-clinical groups (Daalman et al., 2011; Badcock and Hugdahl, 2012a). These differences are of value in distinguishing those who do and do not need professional treatment for their voices, and why, (i.e., in uncovering the different mechanisms involved) (Badcock and Hugdahl, 2012b; Larøi, 2012). A key issue in this regard concerns the emotional response to AVH in these groups.

**Figure 1:**
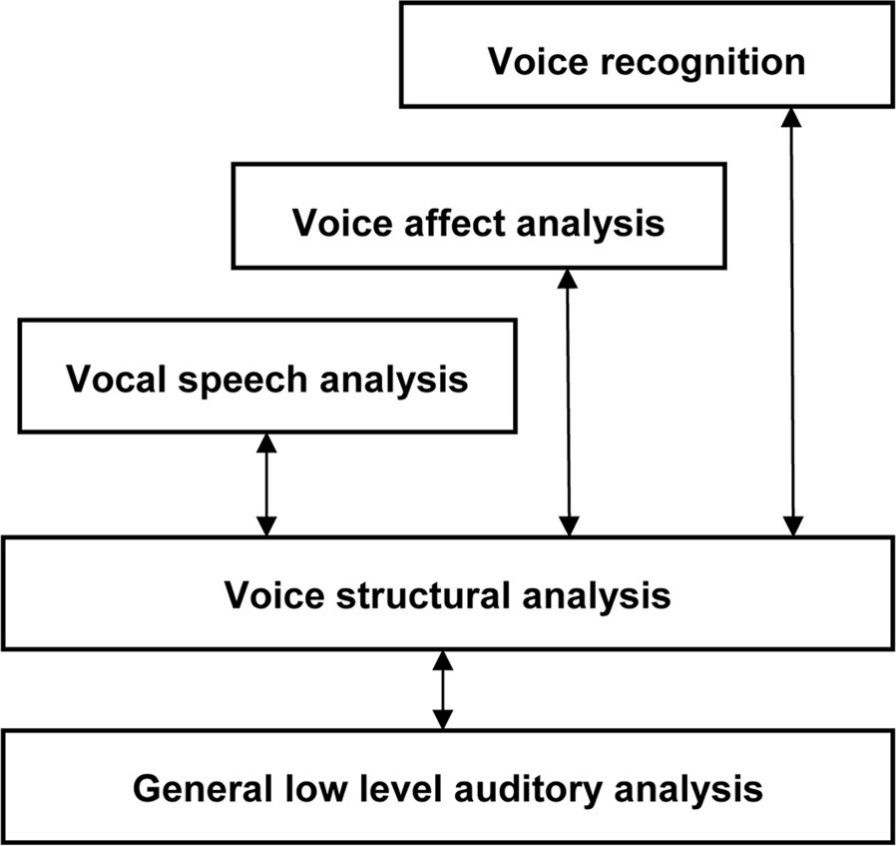
**The “auditory face” model of human voice perception, comprising separable functional pathways for processing speech, affect and identity information in voices [adapted from Belin et al. (2011)]**.

Hallucinated voices in schizophrenia are usually accompanied by significant distress and disruption to daily life (Nayani and David, 1996; Evensen et al., 2011) which often leads voice hearers to seek help for their experiences. In contrast, voice hearing in individuals without a diagnosis of mental illness is more commonly described as being positive, providing a sense of comfort, support or friendship and involving little or no interference to everyday functioning (Andrew et al., 2008; Daalman et al., 2011; reviewed in Lawrence et al., 2010; Hill and Linden, 2013). The reasons underlying these differences in distress between clinical and non-clinical voice hearers are, therefore, clinically significant and appear to be closely tied to how voices are interpreted or appraised (Chadwick and Birchwood, 1994; Garety et al., 2001; Morrison, 2001). In particular, perceptions and beliefs about the identity (e.g., as sounding like the voice of someone other than the self) and interpersonal attitude (power and intent) of hallucinated voices have been shown to be especially important (Nayani and David, 1996; Birchwood and Chadwick, 1997; Mawson et al., 2010). Within this literature, several important points emerge. First, beliefs about the identity and the content of AVH are clearly separable and sometimes incongruent^1^. This observation is consistent with current models of human voice perception and memory (see Figure 1) which show that different types of information (speech, identity, and affect) are processed somewhat independently in the brain (Stevens, 2004; Relander and Rämä, 2009; Belin et al., 2011). Second, beliefs about the identity of hallucinated voices appear to be more decisive in provoking distress than the content of AVH^2^ (Peters et al., 2012; Hill and Linden, 2013). Finally, there is a growing body of evidence which suggests that the voice/voice hearer relationship mirrors “real” social relationships in the voice hearers' daily life (Birchwood et al., 2004; Hayward et al., 2011). Indeed, it has been argued that the defining essence of AVH includes voices with a quality of realness (i.e., distinct from internal dialog) that are assigned a characterized identity (e.g., to a male voice, or a spiritual force), which leads to a relationship with the voice (Beavan, 2011).

These studies highlight the importance of voice identity in distinguishing clinical and non-clinical hallucinations, and raise the possibility that the perception of voice identity in AVH is grounded in the mechanisms of human (i.e., real, external) voice perception. The goal of this review, therefore, is to critically evaluate current evidence on the perception and recognition of voice identity in clinical and non-clinical voice hearers in order to establish any similarities and differences in voice processing ability in these groups. We also aim to expand on the phenomenological description of identity in AVH by drawing on the qualities of real, external voices. The literature in this area is extremely diverse ranging from psychoacoustics to vocal stereotypes but has not previously been integrated with AVH. Here we try to synthesize some of this research to provide a deeper understanding of the features used to judge voice identity in real and hallucinated voices. Whilst we recognize that AVH occur in a range of other disorders, the scope of this review is limited to AVH in people with schizophrenia and in non-clinical (i.e., healthy) comparison groups.

## Perception of voice identity in real and hallucinated voices

### Human voice perception

**Figure 2:**
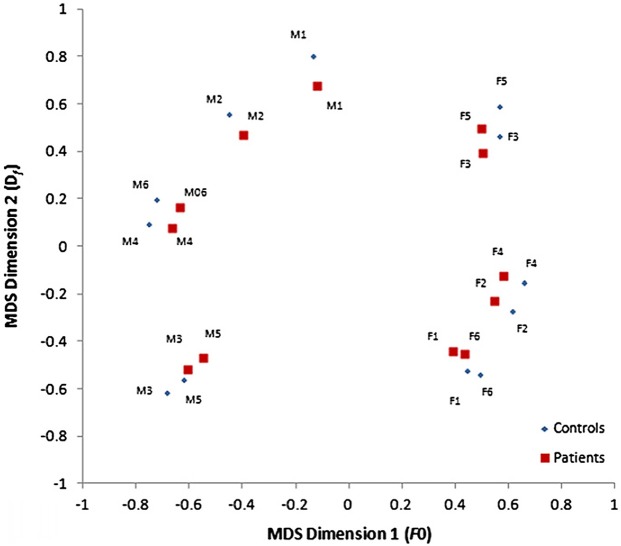
**Two-dimensional voice space derived from multidimensional scaling of voice dissimilarity ratings, defined by the fundamental frequency (*F*_0_) and formant dispersion (*D*_*f*_) of the voice, for healthy individuals and patients with schizophrenia.** Voices that appear more widely separated in this voice space are perceived as more different than those closer together. [Reprinted from Chhabra et al. (2012a) with permission from Elsevier]. Note: *M*, male voices; *F*, female voices.

Everyday social interactions rely heavily on the information conveyed in voice. In fact, the human voice has often been described as an “auditory face” (see Figure 1; Belin et al., 2004, 2011; c.f. Bruce and Young, 1986) since, along with linguistic information, it provides important social information about who you are (speaker identity) and how you feel (emotion). In particular, listeners are generally good at determining the physical characteristics of a speaker from their voice, including their gender (Mullennix et al., 1995; Whiteside, 1998; Sokhi et al., 2005; Pernet and Belin, 2012), approximate age (reviewed in Kreiman and Sidtis, 2011; Zäske and Schweinberger, 2011), size or strength (von Kriegstein et al., 2007; Sell et al., 2010) and attractiveness (Bruckert et al., 2010). For example, Krauss et al. (2002) found that age, height and sex estimated from a two sentence voice sample was only slightly less accurate than that made from a full length photograph. The perception of these physical aspects of identity relies on a variety of low-level acoustic features, including the fundamental frequency (*F*_0_; perceived as voice pitch) and formant frequencies (*F*_*n*_; related to timbre) of the voice (Hillenbrand, 2005; Ko et al., 2006; Latinus and Belin, 2011) which are correlated with speaker size. Consequently, speakers with either lower *F*_0_ or *F*_*n*_ tend to be rated as larger and more masculine and also more attractive, if male, or less attractive, if female (Pisanski and Rendall, 2011). A common approach to examining the variations perceived in voices is to use multidimensional scaling of voice similarity judgments. Participants in such studies listen to a large number of pairs of voices and rate the degree to which the identity of the voices seem similar or dissimilar. What emerges from this approach is that, in fact, different speaker voices can be mapped as individual points within a common two-dimensional “voice space” (see Figure 2 for an example, using data obtained from healthy controls and patients with schizophrenia, Chhabra et al., 2012a) defined by such acoustic characteristics (cf. Baumann and Belin, 2010).

In addition to these physical characteristics, we routinely gain an impression of a speaker's psychological and social identity from the voice alone, including their personality, regional origin (e.g., accent), and socio-economic status (Kreiman and Sidtis, 2011; Hu et al., 2012). Importantly, recent evidence suggests that we automatically evaluate voices along two fundamental dimensions of person perception: warmth and competence (Puts et al., 2007; Ko et al., 2009; McAleer et al., 2010; Teshigawara, 2011). Drawing on the Stereotype Content Model (SCM) of social cognition (Fiske et al., 2007; Fiske, 2012) the warmth dimension captures traits related to perceived intent (trustworthiness, friendliness) whilst the competence dimension reflects traits related to perceived ability (dominance, power). Though the evidence is still accumulating, these two dimensions of voice-based person perception (or vocal stereotypes) are clearly related to the physiologically determined acoustic characteristics of voices noted above (Puts et al., 2007; Wolff and Puts, 2010). For example, Puts and colleagues have shown that lower *F*_0_, formants and formant dispersion (*D*_*f*_) of a voice increases attributions of physical dominance and threat potential among men (Puts et al., 2007, 2012). Social psychologists also argue, however, that warmth and competence judgments are influenced by important social factors, such as perceived cooperativeness and social status cues (Fiske et al., 2007). Thus, individuals high in rank or status are perceived as more competent and powerful than those low in status; though again the *F*_0_ of a voice may play an important role in communicating relative social status between speakers (Gregory and Webster, 1996; Gregory et al., 2001). Importantly, a number of studies have shown that perceptions of warmth and competence from voice also predict people's emotional and behavioral reactions to others, with important social outcomes (Klofstad et al., 2012; Tigue et al., 2012).

Together this literature suggests a biopsychosocial model of human voice perception in which sensory-acoustic and psycho-social cues are combined to: allow the listener to build a representation of speaker identity; discriminate between unfamiliar voices; and recognize familiar speakers—even when they are not in sight (see Belin et al., 2011; Gainotti, 2011; Latinus and Belin, 2011). This model provides a useful conceptual framework to explore the perception of voice identity in clinical and non-clinical hallucinators. To assist in this process a summary of some of the features used to judge voice identity is provided in Table 1. Though not an exhaustive list of features it encourages a detailed comparison (along rows) of identity in real and hallucinated voices, allowing gaps in the phenomenological evidence of AVH to be identified. It also allows a search for any systematic patterns (within columns) of physical and psychosocial features within voice hearing groups that tend to lead to distress.

**Table 1:** **Features of voice identity perceived in real and hallucinated voices**.

**Healthy (non-patient)**	**Clinical AH**	**Non-clinical AH**
**PHYSICAL CHARACTERISTICS**		
Gender	Bias to male voices	No gender bias
Age	Often middle-aged	“–”
Size/strength	“–”	“–”
Attractiveness	“–”	“–”
**PSYCHOSOCIAL CHARACTERISTICS**		
Competence/ability	Dominant/omnipotent	Less dominant
Intent/trustworthiness	Mostly malevolent	More benevolent, neutral
Personality	“–”	“–”
Accent	Sometimes different from voice hearer	“–”
Social status	Voices often judged of higher social rank	“–”
**PERSONIFICATION**		
Human	Real/familiar person	Real/familiar person
	Famous/public figure	Family members
Dehumanized	Robots	Voices of the deceased
Spiritual/supernatural	God, the Devil	Angels, spirits

Note: “–” - no information found.

### Abnormal voice perception

Focusing first on the physical characteristics of AVH, phenomenological surveys show that the perception of gender is a salient feature (McCarthy-Jones et al., 2012). Both male and female voices are heard, however, the former appear to be more common in clinical hallucinators, regardless of the gender of the voice hearer, whereas similar proportions of voice gender are reported by non-clinical voice hearers (Nayani and David, 1996; Stephane et al., 2003; Lawrence et al., 2010; McCarthy-Jones et al., 2012). Since the perception of masculinity, both between and within gender categories (Ko et al., 2009), usually arises from lower *F*_0_ and *F*_*n*_, this difference in gender bias in voice hearers may reflect subtle anomalies in basic sensory processing in clinical hallucinators only (see Badcock, 2010). It must be noted, however, that since this specific proposal has not yet been empirically assessed it is possible that the preponderance of male voices reflects a difference in [cognitive] bias rather than acoustic sensitivity. Nonetheless, subtle shifts toward lower *F*_0_ and *F*_*n*_ would also lead hallucinated voices to sound like an older or stronger speaker. Lending some support to this proposal Nayani and David (1996) observed that hallucinated voices in schizophrenia often sounded “middle-aged” and, more recently, McCarthy-Jones et al. (2012) reported that the majority of clinical hallucinators only heard *adult's* voices. Critically, however, no equivalent data could be found for non-clinical voice hearers, so it is impossible to determine if there are consistent differences across a range of physical characteristics of voice identity between clinical and non-clinical hallucinators. It is important to note, however, that this combination of vocal features (high masculinity, and an older or stronger speaker) would typically be construed as a potential source of threat and could, therefore, contribute to the higher levels of distress associated with clinical AVH. In sum, despite the importance of characterized identity in AVH (Beavan, 2011), many of the physical characteristics of voice identity are under-investigated in studies of either clinical or non-clinical voice hearers. This state of affairs probably reflects a tradition of assessing only a particular set of features in hallucinated voices, together with a lack of suitably refined assessment tools or agreed terminology (Larøi et al., 2012).

Turning next to the psychosocial identity of AVH, following the influential studies of Chadwick and Birchwood (Chadwick and Birchwood, 1994; Birchwood et al., 2000, 2004; Connor and Birchwood, 2012) it is clear that both patient and non-patient voice hearers judge AVH in terms of their power (omnipotence, dominance) and intent (malevolence/ benevolence) which clearly embodies the fundamental dimensions of competence (ability, dominance) and warmth (intent) respectively, perceived in real, external voices (see Table 1). This finding is consistent with the notion that both real and hallucinated voices are constrained by the same underlying mechanisms of interpersonal cognition. Significantly, however, clinical hallucinators are more likely to perceive voices as omnipotent and malevolent compared to non-clinical voice hearers, whose voices are more often judged as neutral or benevolent (Hill and Linden, 2013). The processes underlying this difference are as yet unknown but, drawing from Table 1, could be coupled to the physical characteristics of AVH described above. In addition, the differences in behavioral and emotional reaction to hallucinated voices in clinical and non-clinical voice hearers can be readily understood within the warmth × competence person perception framework described above (Fiske et al., 2007). For example, voice hearers who perceive themselves to be of lower social rank (i.e., less competent) than others^3^ also feel inferior and less powerful than their AVH, and behave accordingly (Connor and Birchwood, 2012; Paulik, 2012; Hill and Linden, 2013): thus, voices perceived as malevolent and omnipotent (i.e., cold and hostile, yet extremely competent) evoke fear and distress and are actively resisted, whilst those perceived to be benevolent (i.e., warm and trustworthy) are engaged with (Sayer et al., 2000; Peters et al., 2012).

Finally, as with real voices, AVH are often recognized as belonging to a particular person (i.e., personified; Stephane et al., 2003; David, 2004). For example, in one recent survey 70% of clinical hallucinators said their voices were similar to those of people who had spoken to them in the past (McCarthy-Jones et al., 2012), though strictly speaking this response might reflect an increased sense of familiarity with a voice, rather than actual recognition of the identity of the speaker. Conversely, in Lawrence et al. data, 70% of non-clinical voice hearers said the identity of their most dominant voice was unknown (Lawrence et al., 2010). Adding further to this issue, Daalman et al. reported similar rates of attribution of identity to a real or familiar person in clinical and non-clinical hallucinators (Daalman et al., 2011), whilst elsewhere it has been reported that patients often identified their AVH as belonging to public/famous figures, rather than the voices of family or friends as reported by non-clinical hallucinators (Leudar et al., 1997; Larøi, 2012). In sum, therefore, there appears to be both differences and similarities in personification between clinical and non-clinical voice hearers—but there is clearly a shortage of direct comparisons of speaker recognition between these groups. Given this limitation, it should be noted that research on beliefs about the origin of AVH may also be informative on personification, since these beliefs refer to identities perceived as real (i.e., human) or not (i.e., dehumanized or spiritual sources). These studies show that dehumanized (e.g., robots, deceased people) and spiritual (e.g., angels, God, the devil) voices occur in both clinical and non-clinical groups (Daalman et al., 2011), consistent with an enhanced perception of agency (competence) and experience (warmth) (cf. Gray et al., 2011), but again with differences in the valence of intent (harmful demons/devils vs. helpful angels/guardians) in those who seek help for their experiences (see Table 1).

What emerges from these comparisons is the extent of the similarity in hearing real and hallucinated voices, as well as some salient differences in the perception of voice identity between patient and non-patient voice hearers. An obvious question therefore arises, namely: do the differences in phenomenology of AVH in clinical and non-clinical voice hearers result from differences in the underlying mechanisms of human voice perception? Consequently, in the following section we provide a summary and critique of several recent studies which have examined the ability to process real, external voices in clinical and non-clinical hallucinators.

## Voice processing abilities in clinical and non-clinical hallucinators

Surprisingly few studies have disambiguated the role of voice specifically in AVH, from that of speech and language activation (see Koeda et al., 2006, for a neuroimaging example of how this can be done). Of those that have examined voice, the vast majority have investigated the processing of emotion in voice (emotional prosody) (Hoekert et al., 2007; Shea et al., 2007; Leitman et al., 2010, 2011; Alba-Ferrara et al., 2012a; Gold et al., 2012; Kantrowitz et al., 2013) rather than the recognition or discrimination of speaker identity. Given the partial segregation of emotion and identity in human voice perception (as shown in Figure 1), it is possible that processing of emotional prosody could be impaired in schizophrenia (as the literature suggests) with the representation of speaker identity being relatively spared. Evidence of such dissociations has previously been observed, for example, in patients with phonagnosia (Garrido et al., 2009; Hailstone et al., 2010). Nonetheless, recent empirical evidence (described below) suggests that this is not the case in individuals with schizophrenia, since evidence is slowly accumulating for a range of difficulties in voice identity processing that may be relevant to, though not necessarily specific for, the experience of AVH. Conversely, processing of voice identity seems to generally intact in non-clinical hallucinators—though as yet, too few studies have been conducted to be certain of these conclusions.

Two recent studies assessed the ability to recognize familiar voices in patients with schizophrenia, with very different methodologies. Zhang et al. (2008) asked schizophrenia patients with and without AVH to classify spoken voices as familiar (e.g., belonging to friends) or unfamiliar (e.g., those of strangers) as part of a neuroimaging study. The results indicated that voice recognition was impaired in patients with AVH compared to healthy controls, which the authors concluded was related to lower activation in the right superior temporal gyrus. Unfortunately, however, signal detection analysis wasn't used, so it is impossible to determine whether these clinical hallucinators had poorer sensitivity to familiar voices or, alternatively, a different response bias (such as a general tendency to classify voices as unfamiliar) compared to controls. In response to these criticisms, Alba-Ferrara et al. (2012b) adopted a signal detection procedure to examine voice recognition in schizophrenia using an established paradigm from the phonagnosia literature, involving presentation of both famous and non-famous voices. In addition to deciding whether the voices heard were famous or not, participants also had to rate the confidence of their responses (remember, know, or guess) and, where possible, recall the name or other details associated with the voice. The results of this more rigorous investigation showed that patients with schizophrenia, particularly those with AVH, performed poorly on this task: that is, they were less sensitive to famous voices than healthy controls, but did not differ in response bias. Thus, there appears to be a link between impaired voice recognition and AVH in schizophrenia (Alba-Ferrara et al., 2012b) which could contribute to the different phenomenological profile of clinical voice-hearers noted above. As noted by the authors, however, the AVH group in this study also rated higher on delusional thinking, and were not significantly different in sensitivity to famous voices than the non-hallucinating patient control group. It is possible, therefore, that voice recognition difficulties contribute specifically to AVH, or alternatively, they may contribute to symptoms that commonly co-occur with hallucinations (such as delusions) or to a broad range of symptoms (including AVH *and* delusions etc.), meriting further investigation. Another possibility is that abnormalities in voice recognition may be a factor that predisposes individuals to hallucinatory experiences, even in the absence of psychosis. Thus, a significant limitation of these previous studies is that they failed to examine the ability of non-clinical voice hearers to recognize external voices.

Our research group has employed a recognition memory task that overcomes this limitation by assessing the ability of non-clinical, as well as clinical, voice hearers to recognize words and voices and integrate this information in memory (Chhabra et al., 2012b). In this study, participants heard two different words spoken in two different voices in sequence, followed—after a brief delay—by a single spoken word probe. The participants had to judge if the probe was a match to one of the study items: that is, to decide if the *combination* of word and voice identity in the probe was exactly the same as one of the first two stimuli. Using signal detection analyses we showed that patients with schizophrenia—both with and without AVH—were impaired at binding words and voices (i.e., remembering who said what) and markedly less accurate in recognizing individual voices, whilst non-clinical voice hearers had no difficulty either binding information or—importantly—in recognizing new words and voices compared to non-hallucinating controls. Though further work is needed to replicate this finding, it suggests a discontinuity in voice recognition difficulties in clinical and non-clinical hallucinators that could flow through to the different characteristics of hallucinated voices in these groups. However, given the lack of specificity to AVH we cannot exclude the possibility that other symptoms of psychosis also arise from abnormalities in human voice recognition.

Whilst we can recognize the voices of people that we know, we can also distinguish new speakers from the features in their voice (see Table 1). Previous literature has shown that this ability to discriminate unfamiliar voices can be dissociated from impairments in voice recognition (Gainotti, 2011); hence clinical and non-clinical hallucinators could share anomalies in voice discrimination even though they differ in vocal recognition. To our knowledge, there have been no direct comparisons of voice discrimination in clinical and non-clinical voice hearers within a single study. However, our research team used an identical voice discrimination task in two separate studies involving patients with AVH and healthy hallucination prone subjects respectively, and found once again that clinical and non-clinical hallucinators differed in their processing of voices (Chhabra et al., 2012a,c). Both of these studies relied on multidimensional scaling (MDS) of voice similarity judgments, since this technique has previously been used to examine how healthy listeners differentiate separate voice identities (Baumann and Belin, 2010).

In the first of these studies (Chhabra et al., 2012a) we asked patients with schizophrenia, with and without AVH, and healthy age-matched controls to rate the degree of dissimilarity between (same sex and different sex) pairs of unfamiliar voices saying the same three-syllable words. A simple MDS solution for the dissimilarity matrices was found, for both patients and controls, with axes corresponding to the *F*_0_ and formant structure (*D*_*f*_) of the voice. This two-dimensional voice space is similar to that described previously by Baumann and Belin (2010)^4^ and suggests that people with schizophrenia represent external voices in a similar way to healthy controls. However, our analyses also showed that both patients groups (i.e., those with and without hallucinations) made significant less use of resonance cues (i.e., *D*_*f*_) to discriminate voices compared to controls (see Figure 2), pointing to some potentially important differences in voice processing abilities in people with schizophrenia. Since subtle alterations in *D*_*f*_ (described above) have been linked to perceptions of masculinity and dominance (Ko et al., 2006; Puts et al., 2007, 2012) one intriguing possibility that emerges from our findings is that anomalies in vocal resonance shape perceptions of power and dominance in AVH and in other symptoms of psychosis (e.g., persecutory delusions). Another intriguing possibility is that the differences in low level acoustic analysis drive the “otherness” or alien quality of hallucinated voices in clinical groups^5^. Though clearly speculative, these proposals may offer new insights into the pathways to psychosis (Smeets et al., 2012) and deserve further investigation.

In the second of our studies we used the same voice similarity judgment task with a group of young adults (undergraduates) who were either predisposed to hallucinate or not (assessed with the Launay Slade Hallucination Scale-Revised; Bentall and Slade, 1985) but had no current, previous or family history of psychosis (Chhabra et al., 2012c). We found the same two-dimensional MDS voice space, defined by *F*_0_ and *D*_*f*_, was used to represent voice identities, as in our first study, but there were no significant differences between high and low hallucination-prone groups. Importantly, the difference in outcome of these studies cannot be due to differences in stimuli or method, since the same task and procedure was used across both. Together our findings indicate that voice discrimination is impaired in clinical hallucinators but intact in non-clinical voice hearers. However, given these data have not yet been replicated, or extended to other types of non-clinical hallucinators (Larøi, 2012), further work will be needed to determine the robustness of our conclusions.

## Summary and conclusions

The phenomenology of identity in AVH is often scantily assessed, limited to the gender or the age of the voice (Larøi et al., 2012; McCarthy-Jones et al., 2012). By drawing on the literature on human voice perception a more comprehensive understanding of identity in AVH can be gained, ranging from the physical characteristics to the psychosocial identity of hallucinated voices. This multifaceted perspective to the perception of voice identity may also be helpful in the development of refined assessment tools for use in clinical practice, or in therapeutic settings aimed at relationships with voices (Pérez-Álvarez et al., 2008; Hayward et al., 2011).

Studying similarities and differences in voice identity perception between clinical and non-clinical hallucinators is also an important issue, with potential implications for early detection of psychosis and/or distinguishing who does/does not need treatment. Significant differences have been shown to be apparent across different levels of identity between clinical and non-clinical hallucinators. The overall profile of more masculine, dominant, powerful, and negatively personified voices in patients with schizophrenia clearly evokes higher levels of distress and, importantly, may point to systematic (i.e., rather than random) changes in perception that require further investigation. Yet recent data shows that psychotic and non-psychotic voice hearers are not easily differentiated in terms of cortical activation (Diederen et al., 2012). There may be many explanations for this discrepancy, but at least one possibility is that the common areas of activation in clinical and non-clinical hallucinators have different causal drivers (Diederen et al., 2012). Moreover, the limited sensitivity in current neuroimaging approaches means that future studies must adopt more sensitive techniques to elucidate the specific neural mechanisms underlying differences in voice identity in clinical and non-clinical voice hearers (cf. Hill and Linden, 2013). Furthermore, this physiological perspective can also now be coupled with the role of social factors in the experience of AVH, providing some interesting new directions for future research. Taking an embodied cognition perspective (Fay and Maner, 2012) for example, do dysfunctional social interactions involve sensory acoustic signals that promote perceptions of ill-intent in both real and hallucinated voices?

Finally, as a result of recent cognitive studies, a major difference in voice processing abilities in patient and non-patient AVH seems to be emerging, in that significant anomalies recognizing and discriminating human voices have been noted in patients with schizophrenia that do not appear to be present in non-clinical hallucinators. Currently, we can only speculate as to whether the observed impairments in voice cognition are directly relevant to the perception of identity in clinical AVH, since research on the processing of real, external voices has proceeded relatively independently from that on phenomenology of AVH. At the same time it seems that poor voice processing skills may be shared with other symptoms of psychosis since neither voice recognition failures nor differences differentiating between unfamiliar speakers were specifically associated with AVH. How the processing of voice identity contributes to other symptoms is unknown, and will require further consideration, though one likely point of convergence is in the experience of paranoia, given that people with persecutory delusions have a tendency to perceive negative intent in others (Combs et al., 2009). In this context, it would be interesting to examine the developmental trajectories of voice processing abilities, since this may be helpful in revealing if there are different functional pathways in clinical and non-clinical voice hearers.

In conclusion, the current evidence suggests there are many similarities in the physical and psychosocial characteristics of real and hallucinated voices—consistent with the notion that AVH are grounded in the mechanisms of human voice perception (Kompus et al., 2011; Aleman and Vercammen, 2012). Indeed, by harnessing current models of human voice perception (Belin et al., 2011) to AVH we may generate more integrated, testable models of hallucinated voiced which go beyond current models of AVH and auditory perception competing for the same speech and language resources, to encompass the wealth of information conveyed in voice (Badcock, 2010; Allen et al., 2012; Hugdahl et al., 2012).

### Conflict of interest statement

The authors declare that the research was conducted in the absence of any commercial or financial relationships that could be construed as a potential conflict of interest.

## Footnotes

^1^For example, voice hearers might hear positive content in their voices yet perceive the intent of the voice as malevolent and untrustworthy.

^2^We do not mean to imply that the content of AVH is unimportant to distress—it clearly is (Beavan and Read, 2010).

^3^For example, due to individual differences in temperament, or early life experiences, such as trauma and abuse.

^4^The stimuli used by Baumann and Belin (2010) were brief vowel sounds, rather than words. Their two-dimensional voice space was defined by the average fundamental frequency (F0) of phonation and the average first formant frequency (*F*_1_).

^5^Thank you to one of the reviewers for this suggestion.
